# Growth inhibition of non-small cell lung cancer cells by AP-1 blockade using a cJun dominant-negative mutant

**DOI:** 10.1038/sj.bjc.6604267

**Published:** 2008-02-19

**Authors:** Y Shimizu, I Kinoshita, J Kikuchi, K Yamazaki, M Nishimura, M J Birrer, H Dosaka-Akita

**Affiliations:** 1 Department of Medical Oncology, Hokkaido University Graduate School of Medicine, North 15, West 7, Kita-ku, Sapporo 060-8638, Japan; 2 First Department of Medicine, Hokkaido University School of Medicine, North 15, West 7, Kita-ku, Sapporo 060-8638, Japan; 3 Cell and Cancer Biology Department, Center for Cancer Research, National Cancer Institute, Rockville, MD 20850, USA

**Keywords:** cJun, AP-1, non-small cell lung cancer, dominant-negative mutant

## Abstract

cJun, a major constituent of AP-1 transcription factor transducing multiple mitogen growth signals, is frequently overexpressed in non-small cell lung cancers (NSCLCs). The purpose of this study is to determine the effects of AP-1 blockade on the growth of NSCLC cells using a cJun dominant-negative mutant, TAM67. Transiently transfected TAM67 inhibited AP-1 transcriptional activity in NSCLC cell lines, NCI-H1299 (H1299), A549 and NCI-H520 (H520). The colony-forming efficiency of H1299 and A549 was reduced by TAM67, while that of H520 was not. To elucidate the effects of TAM67 on the growth of H1299, we established H1299 clone cells that expressed TAM67 under the control of a doxycycline-inducible promoter. In the H1299 clone cells, the induced TAM67 inhibited anchorage-dependent growth by promoting G1 cell-cycle block, but not by apoptosis. The induced TAM67 decreased the expression of a cell-cycle regulatory protein, cyclin A. TAM67 also inhibited anchorage-independent growth of these cells. Furthermore, TAM67 reduced growth of established xenograft tumours from these cells in nude mice. These results suggest that AP-1 plays an essential role in the growth of at least some of NSCLC cells.

c-jun is the cellular homologue of the oncogene v-jun, which was originally identified as the transforming sequence of avian sarcoma virus 17 (Bohmann *et al*, 1987; Maki *et al*, 1987). cJun is also a central component of AP-1, which consists of homodimers and heterodimers of the Jun, Fos and ATF gene family members and regulates transcription through AP-1 and cAMP responsive elements (Angel *et al*, 1987, 1988; Curran and Franza, 1988; Sassone-Corsi *et al*, 1990). Many extracellular stimuli rapidly activate and induce cJun through cytoplasmic signalling cascades to activate cJun target gene transcription (Karin, 1996; Liu *et al*, 1996; Whitmarsh and Davis, 1996).

Although the role of cJun in human cancers remains to be defined, substantial evidence suggests that cJun is involved in cellular proliferation and transformation. Deregulated expression of cJun induces immortalised rat fibroblasts to grow in an anchorage-independent fashion (Schutte *et al*, 1989) depending on the induction of multiple cJun target genes (Kinoshita *et al*, 2003; Leaner *et al*, 2003, 2005; Hommura *et al*, 2004; Katabami *et al*, 2005). cJun is constitutively expressed in rodent fibroblasts transformed by activated c-H-ras and raf-1 (Siegfried and Ziff, 1990; Kolch *et al*, 1993; Osei-Frimpong *et al*, 1994; Rapp *et al*, 1994). A dominant-negative mutant of cJun inhibits the growth and transformation of rodent fibroblasts induced by c-H-ras, c-raf-1 and even by other various types of oncogenes, c-mos, c-myc, c-fos and SV40 T antigen (Rapp *et al*, 1994).

Lung cancer is one of the leading causes of cancer death throughout the world and its cure rate remains dismally low. However, molecular biological studies have demonstrated the existence of multiple genetic and epigenetic alterations of oncogenes and tumour suppressor genes in the process of lung carcinogenesis, providing useful information for new therapeutic strategies. Previous immunohistochemical studies revealed that cJun is highly expressed in 31–50% of non-small cell lung cancer (NSCLC) tissues (Wodrich and Volm, 1993; Szabo *et al*, 1996), while no immunoreactivity was detected in normal lung counterparts. Taken together with its transforming properties, cJun may have roles in lung carcinogenesis and/or lung cancer growth.

Recently, it has been reported that forced expression of cJun increases anchorage-independent growth in a human bronchial epithelial cell line and that constitutive expression of a dominant-negative mutant of cJun inhibits anchorage-independent but not anchorage-dependent growth of a lung cancer cell line (Maeno *et al*, 2006). These findings suggest rather restrictive roles of cJun in the acquisition of anchorage independence in the process of human lung carcinogenesis. In breast and colon cancer cells, however, inhibition of cJun has been shown to induce G1 cell arrest on anchorage-dependent conditions (Liu *et al*, 2004; Suto *et al*, 2004). In addition, its effects on *in vivo* tumour growth of lung cancer cells have not been investigated. The roles of cJun in growth remain to be elucidated in lung cancer, when considering its potential as a therapeutic target.

In the present study, we investigated the effect of a dominant-negative mutant of cJun, TAM67 (Rapp *et al*, 1994), on the growth of NSCLC cells. Transiently transfected TAM67 inhibited colony formation of H1299 and A549 cell lines, two out of the three NSCLC cell lines tested. Induction of TAM67 under the control of a tetracycline-inducible promoter suppressed both anchorage-dependent and -independent growth in H1299 cells by promoting G1 cell-cycle arrest. Furthermore, the induction of TAM67 significantly decreased *in vivo* tumour growth of the cells.

## MATERIALS AND METHODS

### Plasmids

Plasmid pLRT contains all the components of the reverse tetracycline-regulated (rtTA) system (Tet-on system), a drug-selectable marker of blasticidin *S* deaminase as described elsewhere (Watsuji *et al*, 1997). The plasmid pLRT-TAM67 (or -GFP) was constructed by subcloning TAM67 (or GFP) cDNA from pGEM-TAM67 (or -GFP) into the parental pLRT vector (Kinoshita *et al*, 2003).

### Cell lines and culture

The human NSCLC cell lines, H1299, A549 and H520, were cultured in RPMI 1640 medium supplemented with 10% fetal bovine serum (FBS) and 0.03% glutamine at 37°C in an atmosphere of 5% CO_2_.

To establish TAM67- and GFP-inducible H1299 clone cells (H1299 Tet-on TAM67 clone cells and H1299 Tet-on GFP clone cells), pLRT-TAM67 and -GFP were transfected into packaging Phoenix A cells by calcium phosphate transfection, and retroviruses were harvested after 48 h of transfection and infected into H1299 cells as described elsewhere (Watsuji *et al*, 1997). Stable transfectants were selected by blasticidin (Invitrogen, Carlsbad, CA, USA) at 5 *μ*g ml^−1^ and screened by western blot analysis for inducibility of TAM67 expression in response to 2 *μ*g ml^−1^ doxycycline.

### Luciferase assay

To see the effect of transient expression of TAM67, H1299, A549 and H520 cells were plated in 24-well plates and cotransfected with 0.1 *μ*g of TRE2-luciferase plasmid containing two AP-1-binding sites (Leaner *et al*, 2003) and either 0.2 *μ*g of pCMV-TAM67 or pCMV control vector using Fugene 6 (Roche Diagnostics, Basel, Switzerland). To correct for transfection efficiency, 8 ng of renilla-luciferase plasmid was also cotransfected. Transfected cells were lysed 36 h after transfection and luciferase activity was measured using the Dual-Luciferase Reporter Assay System (Promega, Madison, WI, USA). To increase AP-1 activity, cells were treated with 0.1 nM 12-*O*-tetradecanoylphorbol-13-acetate (TPA) for 6 h before harvesting.

To see the effects of induced expression of TAM67, H1299 Tet-on clone cells were cotransfected with 0.1 *μ*g of TRE2-luciferase plasmid and 8 ng of renilla-luciferase plasmid with or without 0.2 *μ*g ml^−1^ doxycycline, and luciferase activity was measured as described above.

### Colony formation assay

The colony formation assay was described previously (Rapp *et al*, 1994). Briefly, H1299, A549 and H520 cells were plated on 60-mm plates and cotransfected with 0.3 *μ*g of pSV2neo containing a neomycin-resistant gene and either 2 *μ*g of pCMV-TAM67 or pCMV. Forty-eight hours after transfection, the cells were trypsinised and equally split into six-well plates. Geneticin (G418; Sigma, St Louis, MO, USA) was added to a final concentration of 200 *μ*g ml^−1^. All cells were found to be killed at this concentration if not transfected with pSV2neo plasmid. After 2 weeks of selection in geneticin, resistant colonies were stained with crystal violet and counted.

### Evaluation of DNA-binding activity of AP-1 by an enzyme-linked immunosorbent assay

H1299 Tet-on clone cells were cultured in 100-mm plates in the absence or presence of 2 *μ*g ml^−1^ doxycyclin for a week and then nuclear extracts were prepared from the cells using the Nuclear Extract Kit (Active Motif, Carlsbad, CA, USA). The DNA-binding activity was quantified by enzyme-linked immunosorbent assay (ELISA) using the TransAM AP-1 family transcription assay kit (Active Motif) as described previously (Debinski and Gibo, 2005; Polytarchou *et al*, 2005). Nuclear extracts were incubated in 96-well plates coated with immobilised oligonucleotide containing a consensus-binding site for AP-1 (5′-TGAGTCA-3′). AP-1 binding to the target oligonucleotide was detected by incubation with primary antibodies directed against cJun, JunB, JunD, cFos, FosB, Fra-1 and Fra-2, visualised with secondary antibody conjugated with horseradish peroxidase and developing solution, and quantified by spectrophotometry at 450 nm with a reference wavelength of 655 nm. Note that the antibody against cJun recognises phosphorylated serine 73 of the transactivating domain of cJun and does not detect TAM67, in which most of the transactivating domain is deleted. Nuclear extracts from K562 cells stimulated by TPA were used as a positive control of the assay for cJun, JunB, JunD, cFos, FosB and Fra-1, and those from WI-38 cells stimulated by TPA for Fra-2. For the competition analysis, nuclear extracts were preincubated with the wild-type or mutated AP-1 consensus oligonucleotide.

### Western blot analysis

Purified total protein (20 *μ*g) was separated by SDS-polyacrylamide gel electrophoresis, transferred to nitrocellulose membranes and reacted with rabbit polyclonal anti-cJun antibody (AP-1/Ab-1; Oncogene Science, Uniondale, NY, USA), rabbit polyclonal anti-cyclin A antibody (sc-751; Santa Cruz Biotechnology, Santa Cruz, CA, USA), mouse monoclonal anti-cyclin E antibody (sc-247; Santa Cruz Biotechnology), mouse monoclonal anti-Kip1/p27 antibody (BD Biosciences, San Jose, CA, USA) and rabbit polyclonal anti-actin antibody (Sigma). The primary antibody was detected using anti-rabbit or anti-mouse antibody conjugated with horseradish peroxidase and visualised using the Amersham ECL system. Band intensities were quantified by densitometry.

### Cell growth assays

Anchorage-dependent growth of H1299 Tet-on clone cells was measured in 96-well plates using an MTT (dimethyl thiozolyl-2′, 5′-diphenylo-2-*H*-tetrazolium bromide) assay (nonradioactive cell proliferation assay; Promega) every other day for 7 days in the absence or presence of 2 *μ*g ml^−1^ doxycycline. To each well was added 15 *μ*l of Dye Solution, and then cells were incubated at 37°C. After 4 h, 100 *μ*l of Solubilization/Stop Mix solution was added to each well. One hour later, the contents of the wells were mixed to yield a uniformly coloured solution. The absorbance at 590 nm was recorded using a 96-well plate reader, and the survival fraction was quantified. Anchorage-independent growth assays of these cells were performed using 0.35% soft agarose (Seaplaque; Cambrex, Rockland, ME, USA) in six-well plates as described previously (Sabichi *et al*, 1998). After 2 weeks of incubation in the absence or presence of 0.1 *μ*g ml^−1^ doxycycline, viable cells were stained by iodonitrotetrazolium chloride (Sigma) and colony numbers were counted.

### Cell-cycle analysis

H1299 Tet-on clone cells were cultured in 100-mm plates in the absence or presence of 2 *μ*g ml^−1^ doxycycline for 4 days. Then, cells were trypsinised, washed twice with PBS and fixed in 70% ethanol at −20°C. Fixed cells were centrifuged and resuspended in 250 *μ*g ml^−1^ RNase and 50 *μ*g ml^−1^ propidium iodide (Sigma) for DNA staining. DNA content was measured by a FACS flow cytometer (BD Biosciences). DNA histograms were obtained using ModFit LT 2.0 (Verity Software House, Topsham, ME, USA).

### Apoptosis assay

H1299 Tet-on clone cells were cultured in 100-mm plates in the absence or presence of 2 *μ*g ml^−1^ doxycyclin for 4 days. Cells were trypsinised, washed twice with PBS, fixed in 1% paraformaldehyde for 15 min on ice and then fixed in 70% ethanol. All cells were processed using the APO-bromodeoxyuridine staining kit (Phoenix Flow Systems, San Diego, CA, USA), and subjected to a flow cytometry-based, modified TdT-mediated dUTP nick end labelling (TUNEL) assay as described previously (Sueoka *et al*, 2000). Briefly, the fixed cells were washed and incubated with DNA labelling solution containing terminal deoxynucleotidyltransferase reaction buffer, deoxynucleotidyltransferase enzyme and bromodeoxyuridine triphosphate (BrdUrd-dUTP). The cells were rinsed before being resuspended with fluorescein-PRB-1 antibody solution and analysed by flow cytometry in the presence of propidium iodide/RNase solution. Analyses of 10 000–20 000 events were done with a FACS flow cytometer (BD Biosciences) equipped with a 488-nm argon ion laser and two software packages: CellQuest 3.1 (BD Biosciences) and ModFit LT 2.0 (Verity Software House). Live gating of the forward and orthogonal scatter channels was used to exclude debris and to selectively acquire cell events. A dual display of DNA area and BrdUrd-dUTP incorporation (FITC-PRB-1) was used to determine the percentage of propidium iodine-stained cells that were apoptotic.

### Nude mouse xenograft experiments

H1299 Tet-on clone cells (1 × 10^6^ cells) were injected subcutaneously into the abdomen of BALB/cA Jcl-*nu/nu* female mice (4 weeks old; CLEA Japan, Tokyo, Japan). After tumours developed and reached the size of 30 mm^3^, the mice were randomised to receive doxycycline-containing (200 *μ*g ml^−1^) or doxycycline-free water. The tumour sizes were measured at intervals of 5 days and tumour volumes were estimated using the following formula: length (mm) × width (mm) × width (mm)/2. To determine inducibility of TAM67 expression in tumours, mice were killed 7 days after the randomisation and local skin tumours were removed. Protein was extracted from the tumours and subjected to western blot analysis as described previously (Hakuma *et al*, 2005). The animal studies conducted have been approved by the Institutional Ethical Board for animal welfare at the Hokkaido University Graduate School of Medicine.

### Statistical analysis

All values are presented as mean±s.d. Statistical significance was determined by Student's unpaired, two-tailed *t*-test.

## RESULTS

### TAM67 inhibits AP-1 activity in NSCLC cells

**Figure 1:**
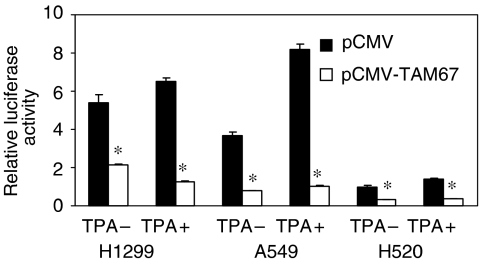
Inhibition of AP-1 transcriptional activity by TAM67 in NSCLC cells. H1299, A549 and H520 cells were cotransfected with 0.1 *μ*g of TRE2-luciferase plasmid and 8 ng of renilla-luciferase plasmid and either 0.2 *μ*g of pCMV-TAM67 or pCMV control plasmid. Transfected cells were lysed 36 h after transfection and luciferase activity was measured. To increase AP-1 activity, cells were treated with 0.1 nM TPA for 6 h before harvesting. The luciferase activity was shown relative to the basal activity in H520 cotransfected with pCMV plasmid without TPA, which was set to 1. Each value represents the mean±s.d. (*n*=3). ^*^P<0.01.

To determine whether TAM67 inhibits AP-1 activity in NSCLC cells, H1299, A549 and H520, were cotransfected with TRE2- and renilla-luciferase vector and either TAM67 expression vector (pCMV-TAM67) or control vector (pCMV). The transiently transfected TAM67 inhibited AP-1 activity in these three cell lines similarly, while basal AP-1 activity in A549 and H1299 was about four- and fivefold higher, respectively, than that in H520 (Figure 1). TAM67 also inhibited TPA-induced AP-1 activity in these cells. These results indicate that TAM67 inhibits AP-1 transcriptional activity in NSCLC cells.

### TAM67 inhibits colony formation of NSCLC cells

**Figure 2:**
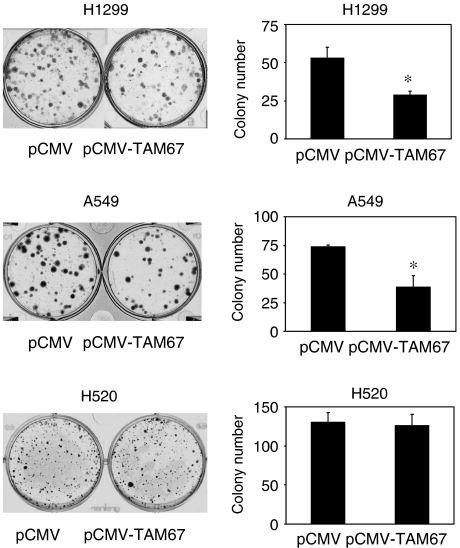
Colony-forming efficiency of TAM67-transfected NSCLC cells. H1299, A549 and H520 cells were cotransfected with 0.3 *μ*g of pSV2neo containing a neomycin-resistant gene and either 2 *μ*g of pCMV-TAM67 or pCMV. After 2 weeks of selection in geneticin, resistant colonies were stained with crystal violet. The number of colonies was shown as the mean±s.d. (*n*=3). ^*^P<0.01.

We next examined whether TAM67 affects colony-forming efficiency. H1299, A549 and H520 were cotransfected with pSV2neo and either pCMV-TAM67 or pCMV. The pSV2neo plasmid but not the pCMV-TAM67 or pCMV plasmid contains a neomycin-resistant gene to select the transfected cells. As shown in Figure 2, the colony-forming efficiency of H1299 and A549 was reduced when these cells were cotransfected with pCMV-TAM67 compared with cells cotransfected with pCMV. On the other hand, colony formation of H520 was not reduced by pCMV-TAM67. These results suggest that AP-1 may play an important role in the growth of H1299 and A549, but not of H520.

Interestingly, some of the colonies of H1299 cells were stained weakly. The weakly stained colonies contained sparse cells, while strongly stained ones had piled-up cells (data not shown). The weakly stained colonies were more prominent in pCMV-TAM67-cotransfected cells than pCMV-cotransfected cells, suggesting that AP-1 may be involved in such neoplastic piled-up phenotype in H1299 cells.

### Establishment of H1299 clone cells expressing TAM67 under the control of a doxycycline-inducible promoter

**Figure 3:**
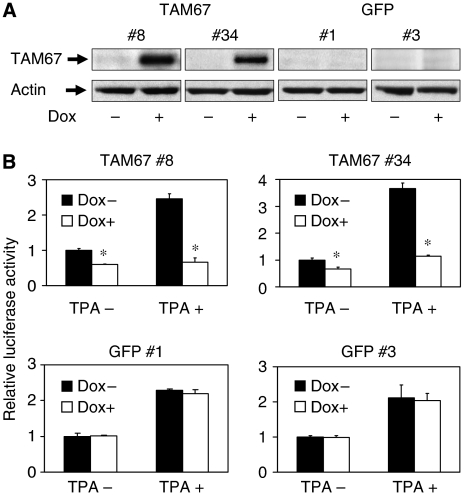
Induction of TAM67 using Tet-on system in H1299 cells and its effect on AP-1 activity. (**A**) TAM67 expression in H1299 Tet-on TAM67 clone cells (TAM67 #8 and TAM67 #34). The cells were cultured in the absence or presence of doxycycline for 48 h, and the extracted cellular protein was examined for the TAM67 expression by western blot analysis. H1299 Tet-on GFP clone cells (GFP #1 and GFP #3) were used as controls. (**B**) Inhibition of AP-1 transcriptional activity in H1299 Tet-on TAM67 clone cells. The cells were cultured in the absence or presence of doxycycline for a week and were cotransfected with 0.1 *μ*g of TRE2-luciferase plasmid and 8 ng of renilla-luciferase plasmid. Transfected cells were lysed 36 h after transfection and luciferase activity was measured. To increase AP-1 activity, cells were treated with 0.1 nM TPA for 6 h before harvesting. Each value represents the mean±s.d. (*n*=3). ^*^P<0.01.

To directly determine the effects of TAM67 on the growth of H1299 cells, we established H1299 clone cells (H1299 Tet-on TAM67 clone cells and H1299 Tet-on GFP clone cells) that express TAM67 or GFP under the control of a doxycycline-inducible promoter. We used a reverse tetracycline-regulated retroviral vector, pLRT-TAM67, that contains a reverse tetracycline-controlled transactivator, blasticidin *S* deaminase domain, and TAM67 or GFP under the control of a tetracycline operator. Stably infected H1299 clone cells were selected by blasticidin and screened for inducibility of TAM67 by western blot analysis. We chose two clones, TAM67 #8 and TAM67 #34, because of their highly inducible TAM67 expression in the presence of doxycycline (Figure 3A). As controls, we used two clones, GFP #1 and GFP #3, which have no inducible TAM67 expression.

We determined if the induced TAM67 inhibits AP-1 transcriptional activity by a luciferase assay. AP-1 activity was inhibited by the induction of TAM67 in the presence of doxycycline in TAM67 #8 and TAM67 #34, especially under TPA stimulation (Figure 3B). On the other hand, AP-1 activity was not affected in GFP #1 and GFP #3. These results demonstrate that the induction of TAM67 can inhibit AP-1 activity in H1299 cells.

### TAM67 affects DNA-binding activity of Jun family

**Figure 4:**
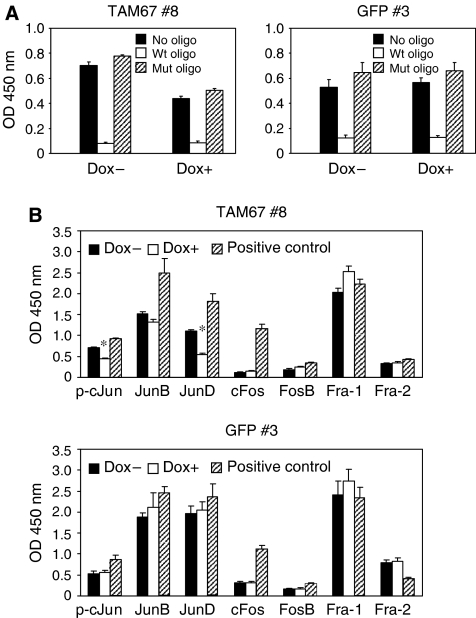
DNA-binding activities of different AP-1 subunits in H1299 cells. H1299 Tet-on clone cells were cultured in the absence or presence of doxycycline for a week and then the nuclear extracts were prepared. DNA-binding activity of each AP-1 family member was measured using TransAM AP-1 kit as described in Materials and Methods. (**A**) DNA-binding activity of cJun. The wild-type (Wt) or mutated (Mut) AP-1 consensus oligonucleotide was used as a competitor for cJun binding. Each value represents the mean±s.d. (*n*=3). (**B**) DNA-binding activities of cJun, JunB, JunD, cFos, FosB, Fra-1 and Fra-2 (data of p-cJun were replotted from Figure 4A). Nuclear extract of K562 cells stimulated by TPA was used as positive control for cJun, JunB, JunD, cFos, FosB or Fra-1 and that of WI-38 cells stimulated by TPA for Fra-2. Each value represents the mean±s.d. (*n*=3). ^*^P<0.01.

We have investigated whether TAM67 modifies DNA-binding activity of AP-1 complexes using the ELISA-based TransAM AP-1 family transcription assay kit (Active Motif). The binding of cJun to AP-1 consensus site was significantly reduced by the induction of TAM67 (Figure 4A and B). The wild-type AP-1 consensus oligonucleotide prevented AP-1 binding to the probe immobilised on the plate, and the mutated consensus oligonucleotide had no effect on AP-1 binding (Figure 4A), confirming the specificity of the assay. In H1299 cells, cJun, JunB, JunD and Fra-1 showed high DNA binding to the AP-1 site, and TAM67 but not GFP significantly decreased the DNA-binding activities of cJun and JunD (Figure 4B). These results suggest that TAM67 may inhibit AP-1 transcriptional activity by modifying the DNA binding of AP-1 components.

### Induction of TAM67 inhibits anchorage-dependent growth by promoting G1 cell-cycle arrest

**Figure 5:**
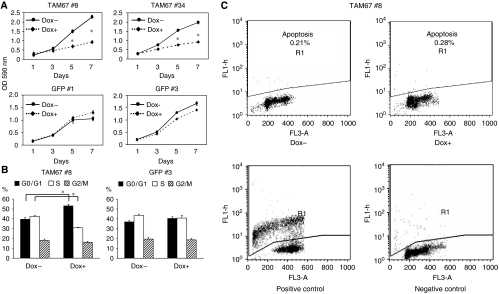
Cell growth assay, cell-cycle analysis and apoptosis assay. (**A**) Inhibition of anchorage-dependent growth by the induction of TAM67. H1299 Tet-on clone cells were cultured in the absence or presence of doxycycline for a week and then cell growth was measured using an MTT assay. Each value represents the mean±s.d. (*n*=4). (**B**) Effects of the induction of TAM67 on cell cycle. H1299 Tet-on clone cells were incubated with 10% FBS in the absence or presence of doxycyline for 4 days. The percentage of cells in each phase was measured by a FACS flow cytometer and analysed using ModFit LT software. Each value represents the mean±s.d. (*n*=5). ^*^P<0.01. (**C**) Apoptosis assay under the induction of TAM67. H1299 Tet-on clone cells were cultured in the absence or presence of doxycycline for 4 days and then apoptosis assay was performed using a flow cytometric analysis as described in Materials and Methods.

We investigated the effects of TAM67 on anchorage-dependent growth using an MTT assay. The growth of TAM67 #8 and TAM67 #34 was suppressed in the presence of doxycycline, but not in GFP #1 or GFP #3 (Figure 5A). We next performed flow cytometric analysis to determine whether TAM67 affects the cell cycle using TAM67 #8 and GFP #3. The induction of TAM67 by doxycycline significantly reduced the percentage of cells in S phase, and increased that in G0/G1 phase in TAM67 #8, while the induction of GFP did not show such effects in GFP #3 (Figure 5B). We further examined whether TAM67 induces apoptosis using TUNEL assay in TAM67 #8. The fraction of DNA fragmentation in the presence of doxycycline remained low at the same level as that in the absence of doxycycline (Figure 5C). These results suggest that TAM67 inhibits cell growth by promoting G1 cell-cycle block in H1299, but not by apoptosis.

### Induction of TAM67 affects the expression of cell-cycle regulatory proteins

**Figure 6:**
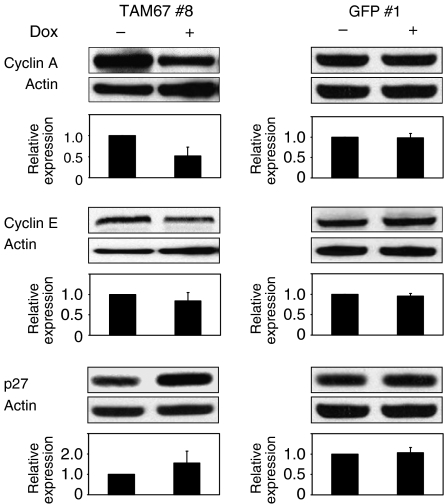
Effects of the induction of TAM67 on the expression of cell-cycle regulatory proteins. H1299 Tet-on clone cells were incubated with 10% FBS in the absence or presence of doxycyline for 4 days. The extracted protein (20 *μ*g) was analysed for cyclin A, cyclin E and p27 expression by western blot analysis as described in Materials and Methods. To normalise the total protein quantity in each sample, the band intensities of cyclin A, cyclin E and p27 were divided by those of actin in the same sample. The expression levels were normalised to those in the absence of doxycycline. Each value represents the mean±s.d. (*n*=5).

We examined whether the induction of TAM67 affects the expression of cell-cycle regulatory proteins. As shown in Figure 6, the induction of TAM67 decreased the expression of cyclin A approximately in half and increased that of p27 slightly. The induction of GFP did not show such effects in GFP #1. The expression of other cell-cycle regulatory proteins, cyclin E, p16 and p21 was not affected by TAM67 (Figure 6 and data not shown). We could not detect cyclin D1 in the cell line.

### Induction of TAM67 inhibits anchorage-independent growth in H1299

**Figure 7:**
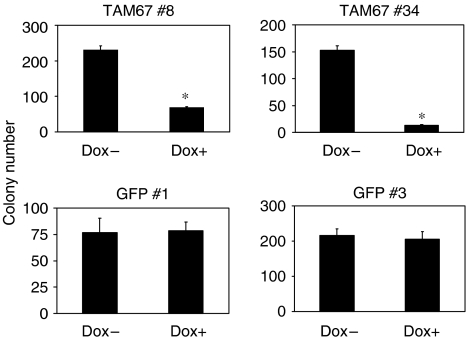
Inhibition of anchorage-independent growth under the induction of TAM67. H1299 Tet-on clone cells were cultured in soft agarose in the absence or presence of doxycycline for 2 weeks. Each value represents the mean±s.d. (*n*=3). ^*^P<0.01.

We determined whether TAM67 inhibits anchorage-independent growth by soft agar assay. As shown in Figure 7, the growth of TAM67 #8 and TAM67 #34 was suppressed remarkably in the presence of doxycycline, but not in GFP #1 and GFP #3. These results indicate that anchorage-independent growth, as well as anchorage-dependent growth, is inhibited by TAM67.

### TAM67 reduces H1299 xenograft tumours in nude mice

**Figure 8:**
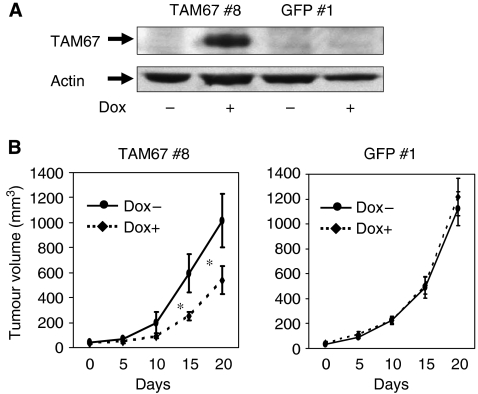
Tumour growth inhibition under the induction of TAM67 in nude mice. H1299 Tet-on clone cells were injected subcutaneously into BALB/c nude mice. After tumours developed and reached the size of 30 mm^3^, the mice were randomised to receive doxycycline-containing or doxycycline-free water. (**A**) Induction of TAM67 expression in established tumours from TAM67 #8 cells. Mice were killed 7 days after the randomisation and local skin tumours were removed. The extracted tumour protein (20 *μ*g) was analysed for TAM67 expression by western blot analysis. (**B**) The tumour sizes were measured at intervals of 5 days and tumour volumes were estimated as described in Materials and Methods. Each value represents the mean±s.d. (*n*=5). ^*^P<0.01.

We investigated whether TAM67 could inhibit tumour growth of H1299 *in vivo*. TAM67 #8 and GFP #1 were injected subcutaneously into nude mice. After tumours developed, the mice were randomised to receive doxycycline-containing or doxycycline-free water. TAM67 expression in the tumours derived from TAM67 #8 was induced when the mice received doxycycline-containing water (Figure 8A). The tumours derived from TAM67 #8 grew fast in the absence of doxycycline (Figure 8B). In the presence of doxycycline, however, the tumour growth was significantly reduced. Similarly, the tumours from GFP #1 grew fast in the absence and presence of doxycycline. These results demonstrate that TAM67 reduces growth of established tumours *in vivo*.

## DISCUSSION

The present study demonstrates the suppressive effects of cJun dominant-negative mutant, TAM67, on both anchorage-dependent and -independent growth of a NSCLC cell line as well as *in vivo* tumour growth. These growth suppressive effects are associated with G1 cell-cycle arrest, suggesting that at least some of NSCLC cells depend on AP-1 for growth.

The observed inhibition of AP-1 activity by TAM67 is consistent with previous reports using various types of cells (Rapp *et al*, 1994; Hennigan and Stambrook, 2001; Ludes-Meyers *et al*, 2001; Suto *et al*, 2004). The decreased DNA binding of cJun and JunD to AP-1 consensus sequence by TAM67 suggests that TAM67 may compete with and replace the proteins in AP-1 complexes to decrease AP-1 activity. On the other hand, TAM67 has been shown to have similar DNA-binding affinity to cJun and to inhibit AP-1 activity through a ‘quenching’ mechanism by inhibiting endogenous Jun and/or Fos proteins (Brown *et al*, 1994). Therefore, there is also the possibility that TAM67 quenches other AP-1 components including Fos proteins.

The colony-forming assay and the subsequent MTT assay demonstrated that the AP-1 blockade by TAM67 inhibits anchorage-dependent growth of H1299 NSCLC cells. The use of multiple TAM67- and GFP-inducible clone cells minimises the possibility of clonal variations and nonspecific effects of doxycycline. Similar effects of AP-1 blockade on growth suppression have been shown in one out of four breast cancer cell lines and in a colon cancer cell line (Ludes-Meyers *et al*, 2001; Suto *et al*, 2004). Taken together, it is suggested that some of cancer cells, including NSCLC cells, depend on AP-1 for anchorage-dependent growth.

Although TAM67 could induce G1 cell-cycle arrest and apoptosis, the results of the apoptosis assay and the observed increase in G1 phase without sub-G1-phase fraction after the induction of TAM67 suggest that the main mechanism of the growth inhibition by the AP-1 blockade is G1 cell-cycle arrest. In fibroblasts, microinjection of antibodies against Jun or Fos proteins blocks entrance into the S phase (Kovary and Bravo, 1991). In a human fibrosarcoma cell line, TAM67 inhibits AP-1 activity and arrests cells predominately in the G1 phase of the cell cycle (Hennigan and Stambrook, 2001). AP-1 blockade in breast cancer cells induces G1 cell-cycle block, which is associated with decreases in G1 cyclin expression and cyclin-dependent kinase activity (Liu *et al*, 2004). Moreover, TAM67 inhibits entrance into the S phase after serum stimulation in colon cancer cells (Suto *et al*, 2004). Taken together, AP-1 blockade inhibits growth by promoting G1 cell-cycle block in various types of malignant cells.

In the present study, we demonstrated that the induction of TAM67 reduce the expression of a cell-cycle regulatory protein, cyclin A. Cyclin A can function during both G1/S and G2/M phases of the cell cycle (Girard *et al*, 1991; Pagano *et al*, 1992; Resnitzky *et al*, 1995). We have previously shown that cyclin A is a direct cJun-target gene in immortalised rat fibroblasts and is necessary for cJun-induced anchorage-independent growth (Katabami *et al*, 2005). The downregulation of cyclin A may also be involved in the mechanisms of G1 cell-cycle arrest induced by AP-1 blockade in H1299 NSCLC cells.

Anchorage-independent growth is a hallmark of cancer and is associated with *in vivo* tumour growth. Suppression of the anchorage-independent growth of H1299 NSCLC cells by AP-1 blockade is consistent with previous reports using another NSCLC cell line (Maeno *et al*, 2006). Furthermore, we demonstrate that *in vivo* tumour growth of the NSCLC cells is also suppressed by AP-1 blockade. *In vivo* tumour growth suppression by AP-1 blockade has also been shown in breast and colon cancer cells (Liu *et al*, 2002; Suto *et al*, 2004). These results suggest that the blockade of AP-1 could be a useful strategy for the treatment of cancers, including NSCLCs.

It has been recently demonstrated that forced expression of cJun increases anchorage-dependent growth in a human bronchial epithelial cell line and that constitutive expression of a dominant-negative mutant of cJun inhibits anchorage-independent but not anchorage-dependent growth of a lung cancer cell line (Maeno *et al*, 2006). Although these findings suggest roles of cJun in the anchorage independence in the process of carcinogenesis, it does not necessarily suggest that cJun is irrelevant to anchorage-dependent growth of NSCLCs. In breast cancer, anchorage-dependent growth is suppressed in one out of four cell lines (Ludes-Meyers *et al*, 2001). In the present study, two cell lines but not the other depend on AP-1 for their anchorage-dependent growth. Therefore, roles of AP-1 in the growth seem to differ among cancer types and even among cell lines in a specific type of cancer.

Different sensitivities to AP-1 blockade by TAM67 among H1299, A549 and H520 may reflect the difference in their basal AP-1 activity. Despite similar suppression of AP-1 activity by TAM67 in these cell lines, the basal activity was higher in H1299 and A549 than in H520, suggesting that the cells with high basal AP-1 activity may be more dependent on AP-1 for their growth. In addition, the compositions of AP-1 dimers may affect the TAM67 sensitivity. Although all three cell lines have high cJun expression, expressions of other components are different; H1299 has high expressions of JunB, JunD, cFos and Fra-1, and A549 has those of cFos and JunD while H520 has minimal or no expression of these genes (Szabo *et al*, 1996). A recent study using several TAM67 mutants that have different binding specificities to AP-1 components indicates that TAM67 likely inactivates Fos family member proteins to suppress cell growth of breast cancer (Lu *et al*, 2005). These results suggest that the basal activity and compositions of AP-1 in cancer cells may affect growth inhibition by TAM67.

In conclusion, we demonstrated that the blockade of AP-1 transcriptional activity by a dominant-negative cJun mutant suppresses NSCLC cells growth both *in vitro* and *in vivo*. These results suggest that AP-1 plays an essential role in the growth of at least some of NSCLC cells.
